# Contrast-enhanced ultrasound (CEUS) of the abdominal vasculature

**DOI:** 10.1007/s00261-017-1329-7

**Published:** 2017-10-05

**Authors:** Vasileios Rafailidis, Cheng Fang, Gibran T. Yusuf, Dean Y. Huang, Paul S. Sidhu

**Affiliations:** Department of Radiology, King’s College London, King’s College Hospital, Denmark Hill, SE59RS London, England, UK

**Keywords:** Contrast-enhanced ultrasound, Aorta, Portal vein, Aneurysm, Endoleak, Trauma

## Abstract

Vascular diseases account for a significant proportion of abdominal pathology and represent a common referral source for abdominal ultrasonographic examinations. B-mode, color Doppler, and spectral Doppler analyses are well-established in the evaluation of abdominal blood vessels although they may occasionally be limited by lower sensitivity for slow flow visualization or the deeper location of abdominal vascular structures. The introduction of microbubbles as ultrasonographic contrast agents has rendered contrast-enhanced ultrasound (CEUS), a valuable complementary ultrasonographic technique, which is capable of addressing clinically significant problems and guiding patient management. The purpose of this pictorial review is to analyze the use of CEUS in the evaluation of abdominal vascular pathology and illustrate such applications by presenting representative images. Pathology discussed includes abdominal aortic aneurysm, post-endovascular treatment aorta, portal vein thrombosis, abdominal vascular trauma, and organ transplantation along with its complications.

**Electronic supplementary material:**

The online version of this article (doi:10.1007/s00261-017-1329-7) contains supplementary material, which is available to authorized users.

Ultrasonography (US) is a well-established first-line modality for the evaluation of abdominal symptoms. Vascular diseases account for a significant part of abdominal abnormalities comprising a wide spectrum of conditions including arterial and venous diseases, diseases affecting native organs, post-operative surveillance and detection of complications, benign and malignant entities, and follow-up of transplantation. Its widespread use is based on numerous advantages, including low cost, repeatability, potential to be performed at any location from the patient’s bedside to the operating room, good patient tolerability, and the absence of contraindications. Nevertheless, US has inherent limitations and in some cases may not successfully address all clinical demands. Inappropriate body habitus, the presence of overlying gas-containing intestinal loops, deep position of abdominal organs, and vascular structures are important limitations for the ultrasonographic evaluation of abdominal abnormalities. When it comes to abdominal vascular diseases, color and power Doppler techniques along with spectral analysis are essential for diagnosis but again have inherent limitations like Doppler angle dependency, limited sensitivity to slow flow, and aliasing or blooming artifact [1]. These limitations are usually accommodated by the performing physician but may hinder proper diagnosis in challenging conditions like the detection of a small or delayed endoleak or the identification of neovascularization within a malignant portal venous thrombus. Computed tomography angiography (CTA) and magnetic resonance angiography (MRA) are currently the reference methods for diagnostic evaluation of abdominal vascular abnormalities, overcoming US limitations, and meeting clinical imaging needs. However, there are situations where CTA and MRA should be avoided, including patients with renal impairment, cardiac pacemakers, and metallic foreign bodies. In a number of patients, US will be the sole imaging modality.

Recent significant technological advances in US with the introduction of elastography and contrast-enhanced ultrasound (CEUS) have expanded capabilities, with the term multiparametric ultrasound (MPUS) used to encompass all the facets of US [2]. Contrast-enhanced ultrasound, using microbubble as ultrasonographic contrast agents (UCA), has gained wide acceptance in many clinical scenarios, culminating in the publication of numerous official recommendations [1]. The recent Food and Drug Administration (FDA) approval for an UCA for characterization of focal liver lesions in adult and pediatric patients is expected to further increase the use of CEUS in the United States [3]. With regard to abdominal vascular pathology, CEUS has been investigated in many applications although considered particularly valuable in the detection and characterization of aortic endoleaks, identification of aortic dissection and rupture, and for differential diagnosis of neoplastic vs. bland thrombus in the portal vein and inferior vena cava (IVC) [1, 4]. Beyond the unenhanced ultrasonographic technique’s inherent advantages previously described, CEUS is also characterized by improved flow visualization even in extremely small-caliber vessels, for example, in tumor neovessels, superior spatial and temporal resolution in real-time evaluation, and increased contrast between blood flow and avascular tissues. This relies on the unique property of UCA to strictly remain within the vascular tree, incapable of diffusion through the vessel wall as their size does not permit this. Moreover, CEUS advantages include dispensing of any prior laboratory tests, excellent safety profile, and limited contraindications [1, 3, 5].

The purpose of this article is to provide an overview of CEUS applications in abdominal vascular abnormalities based on the current literature, and furthermore to present characteristic cases where CEUS proved valuable for diagnosis. The main focus will be in aortic abnormalities including abdominal aortic aneurysm and post-operative surveillance for early detection of endoleaks and venous pathology including neoplastic thrombosis. Less widely performed applications will also be detailed, as summarized in Table 1.

**Table 1:** Summary of CEUS applications for various abdominal vascular systems

Vascular system	Applications	Specific strengths over CTA
Native aorta	Delineation of mural thrombus blood flow within an aneurysm Detection of active extravasation in ruptured AAA Detection of aorto-caval fistulas Detection of aortic dissection	Detection of rupture signs in the emergency department However, an MDCTA should always be performed when available
Post-EVAR aorta	Detection and characterization (classification) of endoleaks Quantification of aneurysm enhancement	Dynamic evaluation Prolonged scanning Better characterization of endoleaks Lack of nephrotoxic contrast agent and ionizing radiation, suitable for long-term follow-up
Portal vein	Improved detection of portal vein thrombus Characterization of portal vein thrombosis as benign or malignant	Increased spatial and temporal resolution within the field-of-view Improved detection of neovessels
Renal arteries	Improvement of renal arteries evaluation with Doppler technique	–
Hepatic/mesenteric arteries	Improvement of mesenteric artery evaluation	–
Trauma	Detection of parenchymal injuries Detection of vascular pathology like pseudoaneurysm or active bleeding	Real-time evaluation Prolonged continuous scanning
Transplantation	Detection of vascular complications like hepatic artery and portal vein thrombosis or stenosis	Real-time evaluation Prolonged continuous scanning

## Technique and safety

CEUS is performed with the intravenous administration of a bolus dose of the UCA, and essentially always performed after a complete unenhanced ultrasonographic examination. This allows the examiner to identify the area of interest, establish an initial opinion, ascertain the viability of a subsequent CEUS examination and plan the procedure to maximize the diagnostic outcome. Once the unenhanced ultrasonographic protocol is complete, having appreciated the gray-scale, color, power Doppler, and spectral analysis findings, an intravenous catheter can be placed in the antecubital fossa. It is best to insert the intravenous catheter following the baseline US to avoid unnecessary cannulation if the CEUS examination is not deemed useful. In general, the amount of UCA administered varies depending on the ultrasound machine’s sensitivity and the product used. SonoVue™ (Bracco SpA, Milan, Italy) is the most widely used contrast agent in Europe and consists of microbubbles containing an inert gas (sulfur hexafluoride) encapsulated by a phospholipid shell, marketed as Lumason™ (Bracco SpA, Milan, Italy) in the United States. A dose of 2.4 mL of Lumason™/SonoVue™ per injection is considered adequate for the liver and other abdominal vascular procedures. A second dose of 2.4 mL can be administered if needed. UCA are strict intravascular agents, large enough (10 μm) to preclude passage through the vascular endothelium, but small enough to circulate through small capillaries. Crucially, the metabolism of UCA renders them independent of renal excretion, the phospholipid shell is metabolized by the liver and the contained inert gas is exhaled by the lungs. As a result, CEUS can be safely performed in patients with renal impairment. In order to achieve optimal visualization of the UCA, a contrast-specific ultrasonographic technique should be applied. Pulse inversion and amplitude-modulation techniques which in general suppress echogenic signals originating from static tissues while visualizing echogenic signals produced by oscillating microbubbles are used. This results in the optimal echogenicity distinction between UCA and static tissues and offers the best spatial and temporal resolution. Two valuable techniques in vascular CEUS include the replenishment mode after a high-Mechanical Index pulse and the Temporal Maximum Intensity Projection (MIP) mode. In the first technique, a high-MI ultrasound pulse is used to disrupt all the microbubbles lying within the imaging field with replenishment allowing observation of the enhancement pattern of structures. In the second technique, the ultrasound device aggregates bright echoes of the UCA and creates cumulative images which illustrate the vascular pattern or architecture of structures under investigation [1, 3, 4, 6].

Among its advantages, CEUS can be performed without any prior laboratory examination as impaired renal function is not a contraindication for administration of UCA, contrary to CTA and MRA. The contraindications for CEUS are limited and include known history of allergic reaction to the UCA itself, severe pulmonary hypertension and pregnancy. The contraindication of right-to-left shunt has been recently discontinued [1, 7]. SonoVue™ has been extensively investigated for adverse reactions and has an excellent safety profile. Serious adverse reactions occurred in only 0.0086% of patients and treatment was necessary in only four patients. This adverse reaction rate is considered comparable to the rate of MR contrast agents and lower than CT contrast agents [1, 8, 9]. CEUS is a safe technique; however, given the very small likelihood of adverse reactions, resuscitation equipment should be available in every US Department where CEUS examinations are performed.

## Clinical applications

### Native aorta

The term abdominal aortic aneurysm (AAA) refers to an irreversible enlargement of the abdominal aorta of more than 3 cm or 50% of reference diameter [10]. US is excellent for screening or diagnostic evaluation and follow-up of AAA with high sensitivity and specificity and excellent intra- and inter-observer agreement [11]. The use of UCA adds little to the evaluation of an uncomplicated AAA, although it will readily and accurately delineate mural thrombus and differentiate this from slow blood flow, often not visualized with conventional US techniques [1, 4, 12, 13]. Rupture of an AAA; associated with high mortality, necessitates early and accurate diagnosis with immediate treatment [10, 14]. Rupture risk increases with increasing aneurysm diameter rising to > 30% for aneurysms larger than 7 cm [10]. Patients presenting with abdominal pain of acute onset and low blood pressure or decrease of hematocrit may signify an AAA rupture, and US can exclude the presence of an AAA. US has limited accuracy for detection of rupture, [15]. The use of UCA significantly increases the sensitivity for detection of several findings of AAA rupture. With the intravascular nature of the UCA, CEUS is able to visualize active extravasation and dependent pooling of the UCA in the retroperitoneum or peritoneal cavity. Although these findings closely correlate with those provided by CTA, CEUS has the potential to be performed at the bedside in the Emergency Department, prompting accurate diagnosis with earlier treatment [16, 17].

AAA rupture may rarely be complicated by the formation of an aorto-caval fistula, which needs specific management. Although CTA is the reference method for the evaluation of aorto-caval communications, CEUS has the potential to delineate such communication with high accuracy in a real-time and dynamic manner [18, 19]. Arterial-venous communications have also been demonstrated with UCA in different vascular systems including the femoral vessels [4].

Dissection usually affects both thoracic and abdominal aorta, with isolated abdominal aortic dissection being rare [20]. Symptoms like asymmetric blood pressure, pain of acute onset, and signs of organ dysfunction secondary to ischemia should point toward the diagnosis of aortic dissection [21, 22]. CTA remains the primary modality for emergency evaluation of the whole aorta including the iliac arteries. If US findings are inconclusive but suspicious of dissection, UCA can be used. Suspicious conventional US findings include an intimal flap and bidirectional color flow signals within the lumen of aorta. The administration of UCA readily and accurately visualizes intimal flaps, establishing the diagnosis of dissection. Moreover, CEUS helps identify re-entry points and discriminate true and false lumen as the enhancement of the former precedes that of the later [13, 19, 23–25].

### Post-operative aorta

**Fig. 1:**
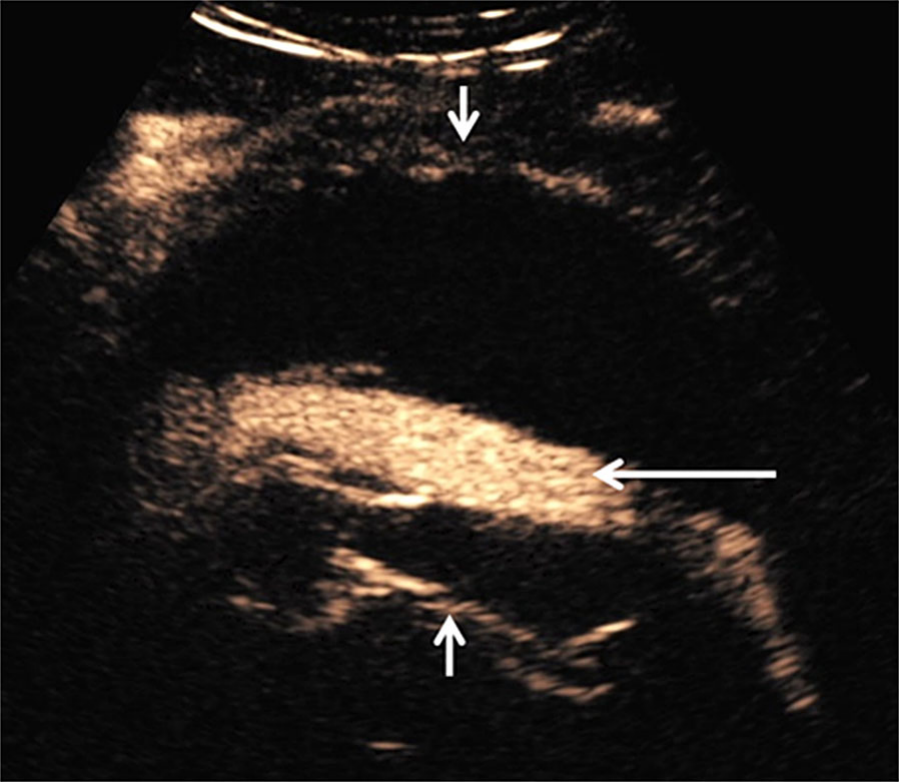
Routine post-elective EVAR follow-up scan from a 83-year-old man showed an expanding aneurysmal sac. CEUS was performed to look for endoleak. Longitudinal view of an aortic stent graft within the dilated aneurysm sac (between short arrows). CEUS image demonstrates microbubble ultrasound contrast within the patent aortic stent graft with no evidence of “endoleak” (long arrow)

An endovascular approach using vascular stents (Endovascular Aneurysm Repair, EVAR) is largely replacing the traditional open surgical repair of AAA. The endovascular approach needs lifelong imaging surveillance allowing for early detection and management of complications. EVAR complications include endoleak, fractures, graft migration, graft disconnection, or progressive enlargement of the aneurysmal sac. Imaging surveillance is routinely performed with CTA or US and is advocated lifelong although increased risk for rupture occurs for the first two to three years after EVAR [26–30]. US is limited by low sensitivity for flow visualization while CTA is disadvantaged by iodinated contrast medium and ionizing radiation. CTA provides “snapshots” of blood flow within a stented aorta, whereas CEUS is characterized by increased sensitivity compared to US, with continuous scanning of the aneurysmal sac in a dynamic and real-time pattern, for > 3 min (Fig. 1). This is useful for characterization of both fast-flowing and slow-flowing endoleaks. With real-time visualization and the option to disrupt the UCA and observe the replenishment pattern, CEUS can accurately characterize the origin of the endoleak, direction, and extent; information essential for type differentiation [4, 31].

**Fig. 2:**
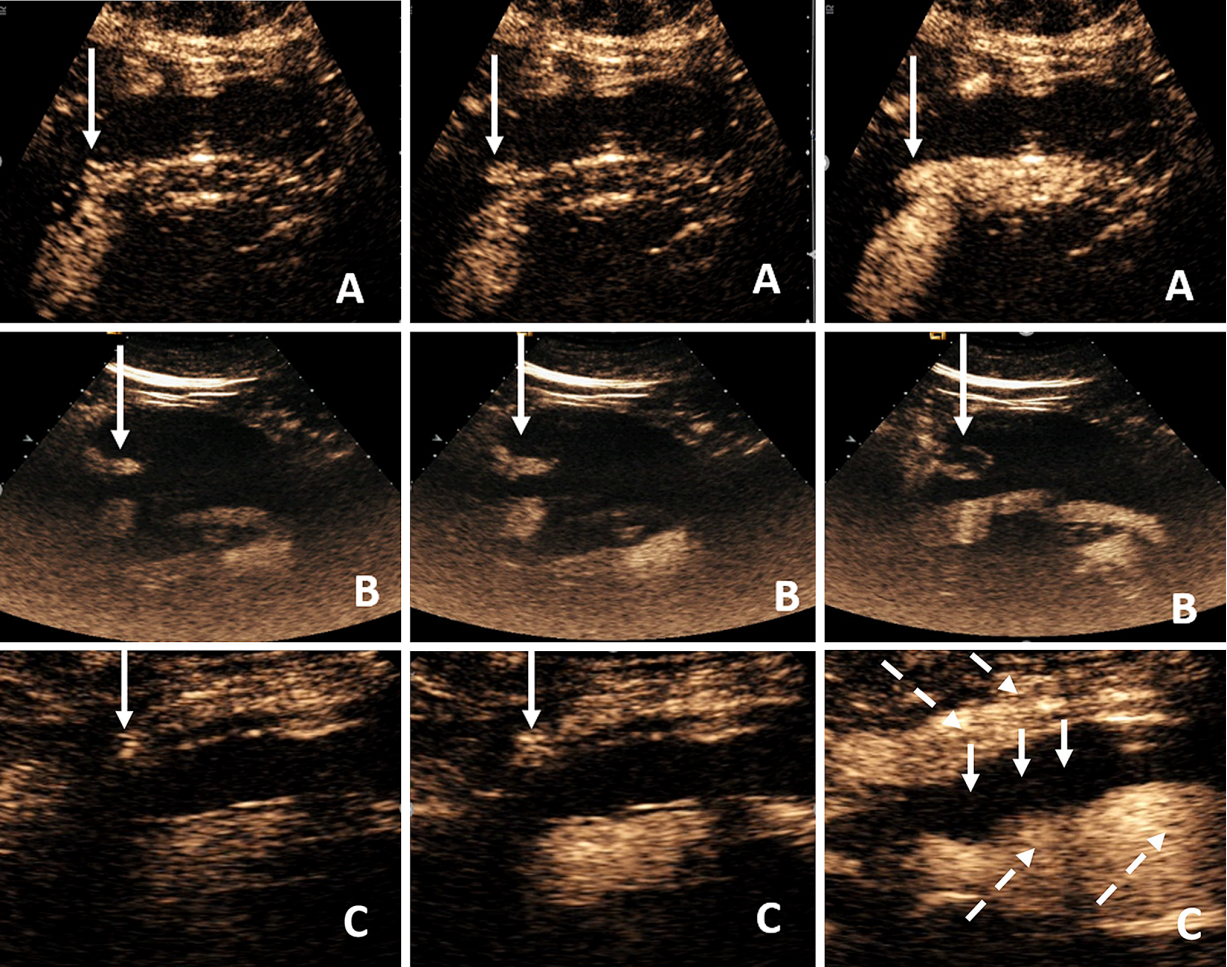
Longitudinal views of aortic stent grafts demonstrates type 1 (row **A**), type 2a (row **B**), and type 3 (row **C**) endoleaks. Each row consists of sequential CEUS images (left to right) demonstrating UCA jets (long arrows) originating from the aortic stent grafts from ineffective proximal seal (row **A**, type 1), persistent filling of the aneurysmal sac from the inferior mesenteric artery (row **B**, type 2a), and inadequate sealing of the overlapping main aortic body and iliac stent (row **C**, type 3). The aneurysmal sac is filled with UCA (broken arrows) with central thrombosis (short arrows)

**Fig. 3:**
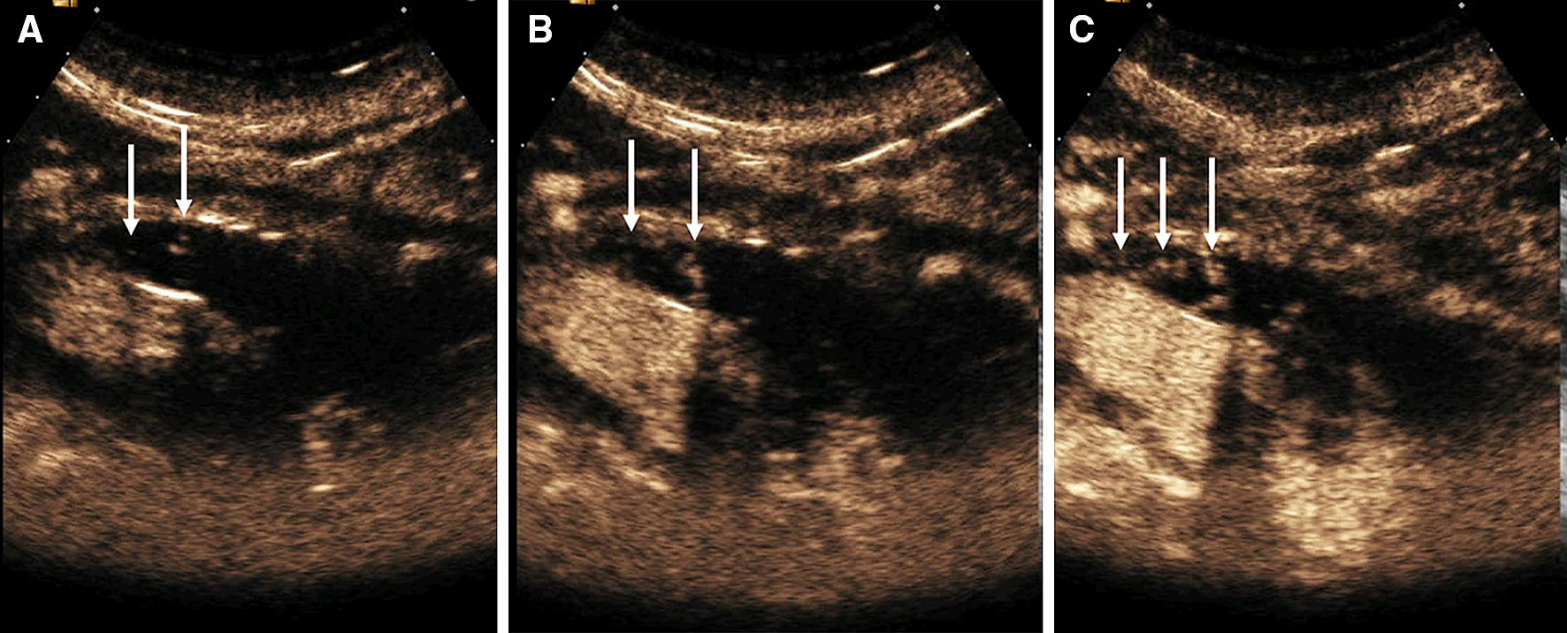
Longitudinal views of aortic graft. Sequential CEUS images (**A**–**C**) demonstrates UCA jets (arrows) originating through the stent graft resulting in a type 4 endoleak due to porosity of the stent graft fabric

Endoleaks represent the presence of blood flow within the aneurysmal sac but outside the stented vessel lumen and characterized based on the direction of blood flow into five categories [26, 29, 32]. Type 1 endoleak refers to an endoleak originating from the attachment of the stent graft with the aortic wall; being proximal (type 1A) or distal (type 1B) attachment. Type 2 endoleaks are the most frequent and represent retrograde blood flow from an anastomotic branch of the aorta or iliac arteries into the aneurysmal sac. Such anastomotic branches are usually the inferior mesenteric or the lumbar arteries. If only one vessel is leaking, the endoleak is classified as type 2A, whereas if multiple vessels are implicated, the endoleak is classified as type 2B. Even though this endoleak usually resolves spontaneously, increased awareness is necessary as increased blood flow and pressure may lead to aneurysm sac enlargement and eventually rupture [26, 29, 30]. Structural failure of the stent may lead to type 3 endoleak, which describes blood flow originating from a defect in the stent, the frequency of this endoleak being proportionate to the stent’s age [26, 29, 33] (Fig. 2; Online Resources 1, 2, 3). Type 4 endoleaks results from porosity of the stent wall, immediately after stent placement or up to 30 days after intervention (Fig. 3). Type 4 endoleaks constitute a diagnosis of exclusion, will usually resolve with normalization of coagulation parameters; carrying no clinical consequence. However, careful characterization is needed as this may mimic other types of endoleaks [26, 29]. The term endotension refers to the enlargement of aneurysm sac without a detectable endoleak, found after a successful EVAR. Endotension is also characterized as type 5 endoleak and is considered to be caused by continuously increased blood pressure within the aneurysm stent [26, 29].

US offers a cost effective, well-tolerated option for imaging surveillance of the post-EVAR aorta, but limited by body habitus, operator experience, and technical artifacts. The diagnostic accuracy varies; studies reporting a 45% positive predictive value and 86% sensitivity for endoleak detection [34] with US detecting more endoleaks requiring intervention compared to CT, with a 90% sensitivity and 81% specificity [35], compared to color Doppler with a sensitivity of 33%–63% and specificity of 63%–93% [36, 37].

CEUS has been widely investigated for accuracy in detecting endoleaks. Optison™ (Mallinckrodt, St Louis, Mo) was found to accurately classify endoleaks as type 1 or 2, enabling US to detect more endoleaks than delayed-phase CTA [38]. The diagnostic accuracy of CEUS with Optison™ for the diagnosis of endoleaks is reported at a sensitivity of 100% and specificity of 65% [39]. CEUS with SonoVue™ has demonstrated variable results, with a sensitivity of 80%–100% and a specificity of 82%–100% in diagnosing endoleaks, outperforming color Doppler US [36, 37, 40–42]. Some studies have concluded that CEUS may even outperform CTA, the current gold standard for evaluation of endoleaks, primarily attributable to the dynamic and real-time nature of imaging [42]. According to a meta-analysis, CEUS pooled sensitivity and specificity for diagnosis of endoleak is 91.4% and 78.2%, respectively, although significant heterogeneity of studies was noted, potentially limiting the accuracy for specificity [43]. Beyond subjective assessment of endoleak presence, CEUS also provides the potential for objective quantitative analysis of aneurysmal sac enhancement. Studies using time–intensity curves have demonstrated that CEUS is 99% sensitive and 93% specific for detection of endoleaks, compared with CTA, with a significant difference between the enhancement level of aneurysms with and without endoleak [44]. If a four-dimensional technique is applied, CEUS has equivalent accuracy to CTA for evaluation of post-operative aortic aneurysm diameter, volume, and endoleak detection in patients with fenestrated endografts [45]. According to a systematic review, CEUS and MRA have superior diagnostic accuracy compared to CTA for identification of post-EVAR endoleaks, although being equivalent to CTA for characterization of endoleaks type 1 and 3 [46]. CEUS was also found to outperform CTA for the diagnosis of delayed type II endoleak [43].

In conclusion, CEUS offers a beneficial alternative to CTA especially for patients with impaired renal function. Moreover, CEUS is also a suitable alternative for younger patients with EVAR reducing the cumulative exposure to ionizing radiation, with the need for lifelong imaging surveillance with CTA. CEUS could be incorporated in diagnostic algorithms for the detection of endoleak as a second step after initial US examination, in order to increase the technique’s diagnostic accuracy. In cases of negative results, the patient could be safely discharged and referred for follow-up imaging. Further imaging with CTA could be reserved for cases with positive results or continued suspicion of endoleak [28, 31, 36, 47].

### Portal vein thrombosis

**Fig. 4:**
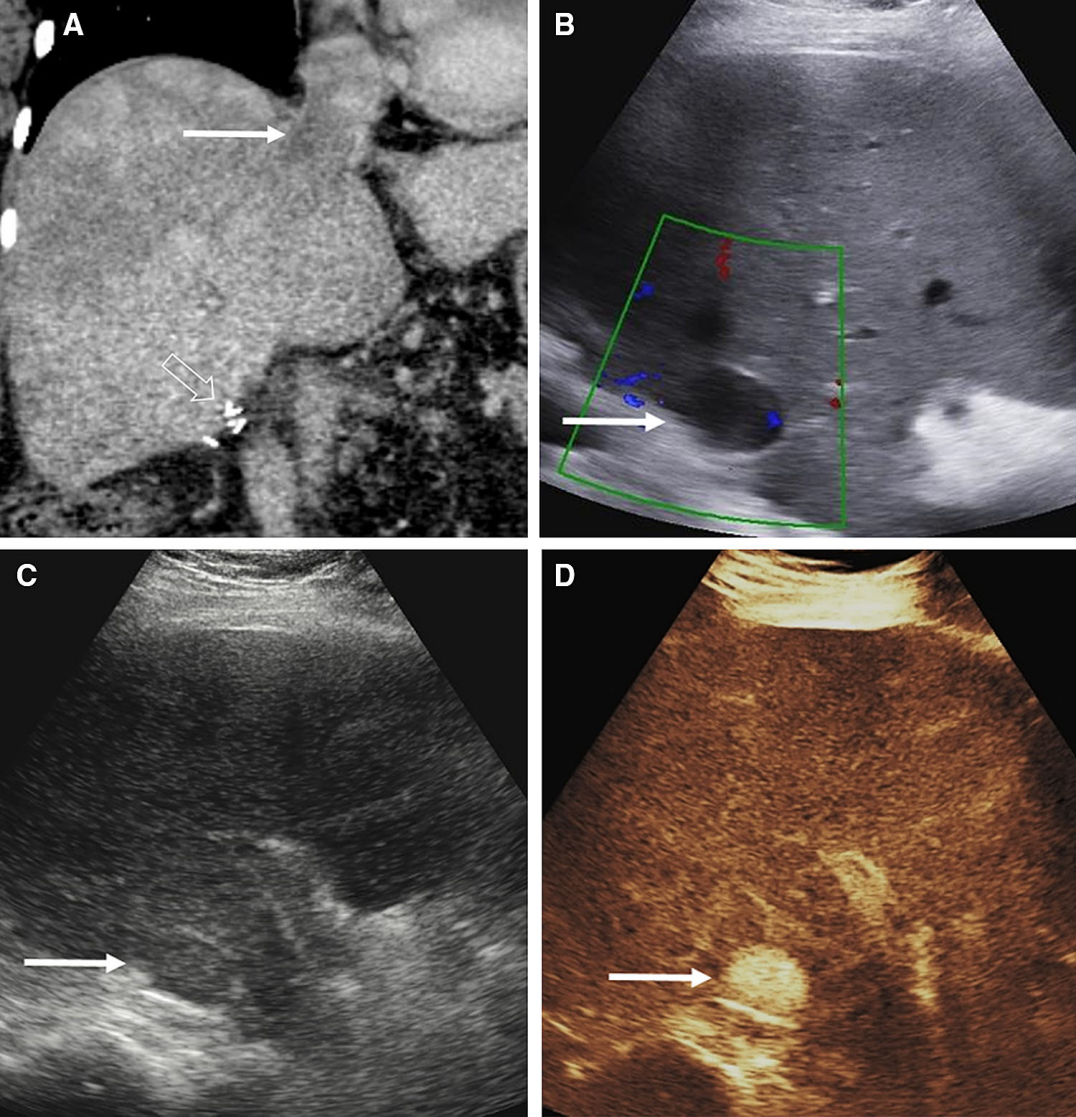
Patient with history of invasive retroperitoneal sarcoma compressing on IVC which was resected (surgical clips, open arrow). She subsequently presented with bilateral lower limb pitting edema. Imaging studies were performed to exclude IVC thrombus (**A**) coronal CECT (**B**) Color Doppler US and (**C**) Gray-scale US demonstrate near occlusive thrombus within the IVC (arrows). (**D**) The thrombus shows enhancement with microbubble ultrasound contrast imaging in keeping with tumor thrombus (arrow)

**Fig. 5:**
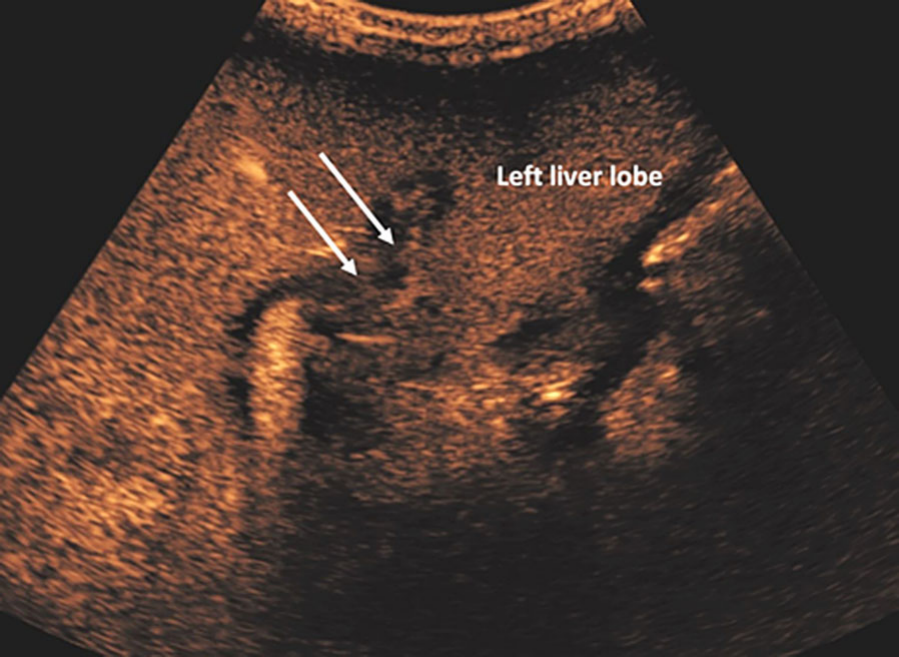
CEUS image demonstrates non-occlusive bland thrombosis of the left portal vein (arrows)

**Fig. 6:**
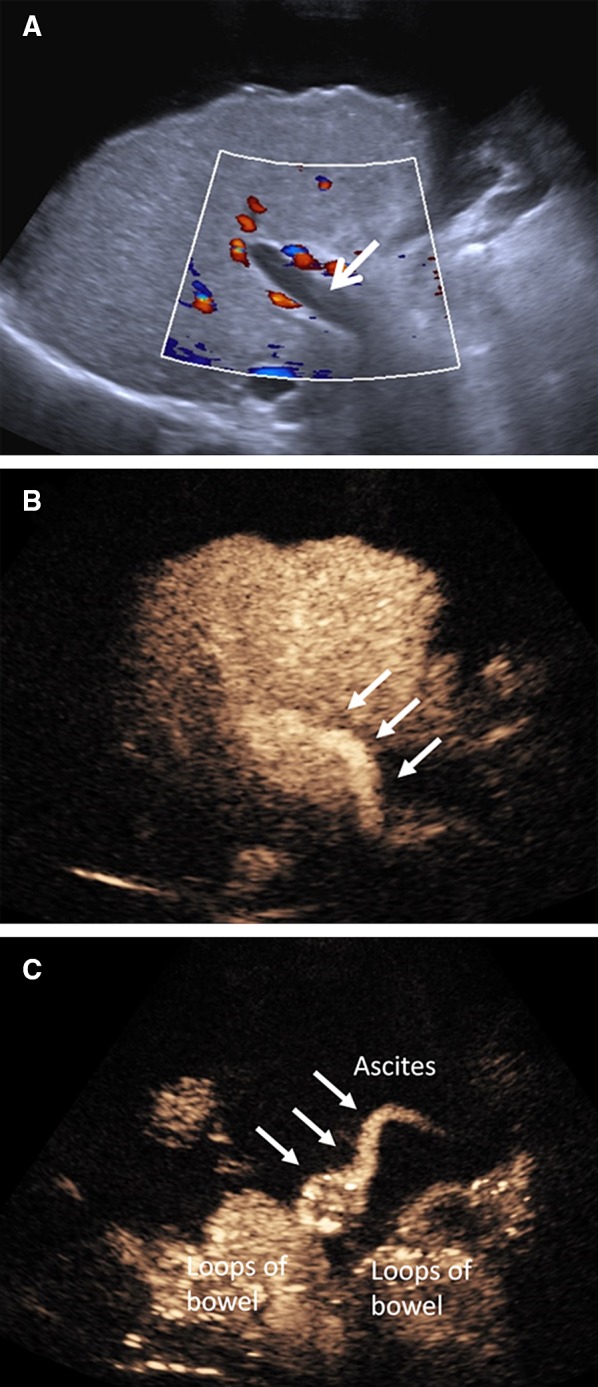
A 66-year-old male with liver cirrhosis developed hepatorenal syndrome. Color Doppler US failed to demonstrate the presence of portal flow likely due to slow flow (**A**, arrow). CEUS was performed instead of CECT due to poor renal function. CEUS demonstrated a patent portal vein (**B**, arrows). Isolated CEUS image from the same patient shows recanalisation of large umbilical vein (**C**, arrows) surrounded by large volume ascites (black area) in keeping with established portal hypertension

Portal vein thrombosis may be bland or neoplastic, in patients with hepatocellular carcinoma and cirrhosis. Accurately characterizing portal vein thrombus as neoplastic or bland is of clinical significance as the former constitutes a contraindication for liver transplantation. Characterization of thrombus as neoplastic can be established on the presence of neovascularization within the thrombotic material. Benign thrombosis manifests with shrinkage of the thrombus or recanalization of the portal vein, seen with color Doppler US, on follow-up examination, whereas increase in thrombus size, disruption of the vessel wall, and infiltration of the adjacent liver parenchyma is in keeping with malignancy [48]. B-mode US is useful in detecting portal vein thrombosis but is unreliable in differentiating benign from malignant thrombus. Color Doppler US can be useful by demonstrating color flow signals within the thrombus although less effectively for small thrombus. A deep location of the portal vein and a potentially unfavorable body habitus may limit sensitivity for detection of neovascularization. CEUS is superior to color Doppler technique for the diagnosis of neoplastic portal vein thrombosis in patients with liver cirrhosis. CEUS has the ability to visualize pulsatile enhancement of portal vein thrombus during the arterial phase, preceding portal lumen enhancement, representing malignant neovascularization [49–54]. CEUS provides conclusive outcomes in > 97% of patients examined, with no further imaging required [51]. In terms of diagnostic accuracy, CEUS was found to be 100% sensitive, 66.7% specific, and 93.3% accurate in the diagnosis of malignant portal venous thrombosis [48]. With the introduction of CEUS in the diagnostic strategy of malignant portal vein thrombosis, fewer interventional fine-needle biopsies are required, which is considered the reference method, and less well-tolerated by the patients [53]. CEUS can also be used to characterize thrombus within other abdominal veins like the hepatic and inferior vena cava (Fig. 4). Moreover, it can accurately detect non-occlusive portal vein thrombus (Fig. 5) and establish the patency of portal vein in cases with slow flow not visualized on color Doppler technique (Fig. 6).

### Abdominal trauma

CEUS in abdominal trauma has been evaluated with promising results. Traumatic parenchymal lesions appear as non-enhancing hypoechoic areas and showing variance with the otherwise normally perfused parenchyma. CEUS can readily identify and characterize lacerations, contusions, and intra-parenchymal or sub-capsular hematomas affecting all solid organs of the abdominal cavity. Based on studies comparing US and CEUS with CT as the reference standard in patients sustaining blunt abdominal trauma, CEUS was found to outperform US in terms of sensitivity and specificity for the diagnosis of solid organ injury, with CEUS demonstrating 69% sensitivity and 99% specificity for diagnosing renal trauma, 84% sensitivity and 99% specificity for liver trauma, and 93% sensitivity and 99% specificity for splenic trauma [1, 55, 56].

**Fig. 7:**
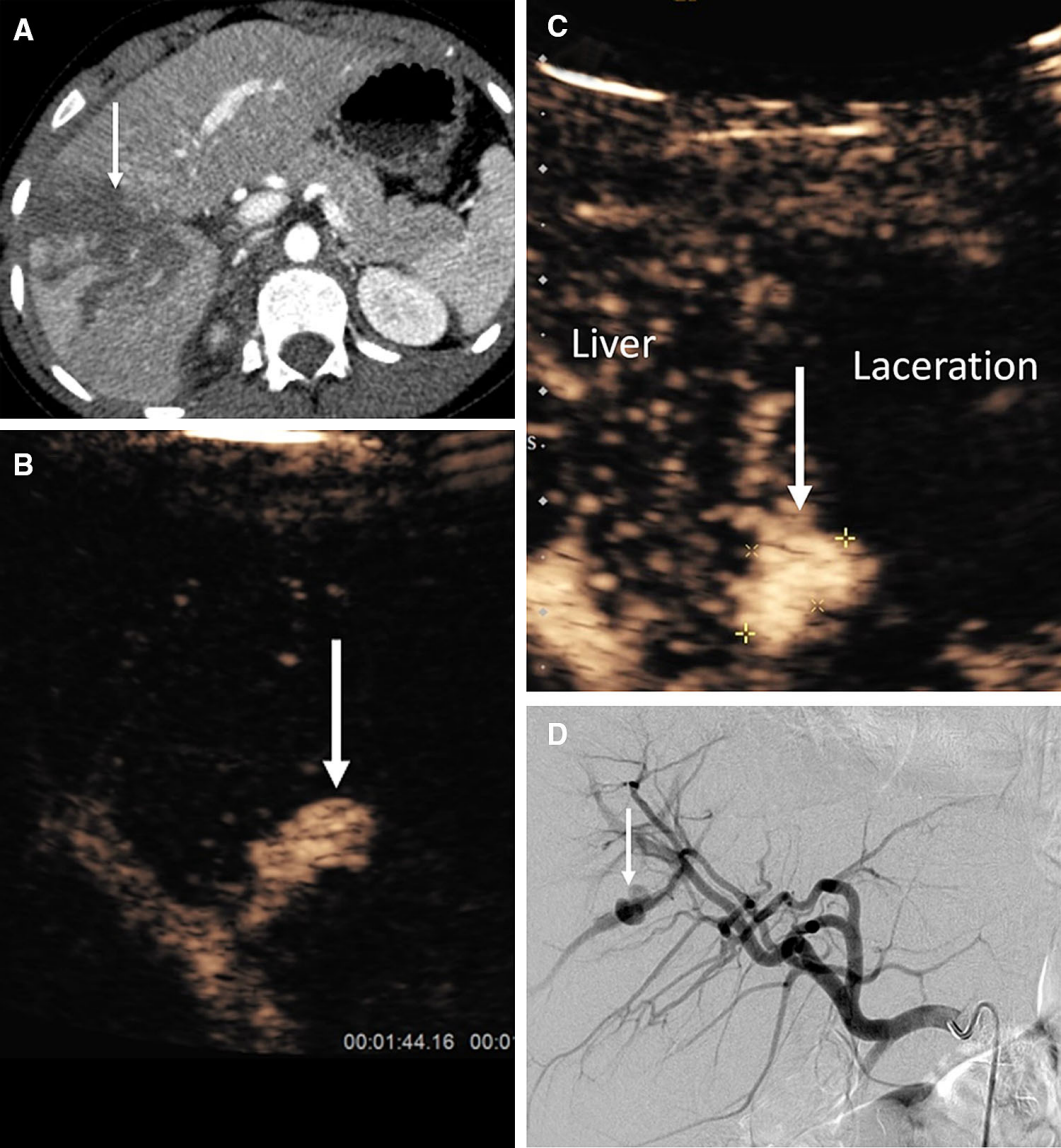
A 14-year-old boy fell downstairs and sustained a grade 4 liver laceration. CECT (axial) shows linear area of liver laceration (**A**, arrow). CEUS of the liver was performed 5 days post-trauma to evaluate the injury. Sequential CEUS images (**B** and **C**) demonstrate a pseudoaneurysm (arrow in **B**, between cursors and arrow in **C**). Image **B** is taken during arterial phase akin to the CT angiographic phase with microbubble contrast only seen in the hepatic artery and pseudoaneurysm. The later image **C** shows the presence of microbubble contrast within the liver. The part of the liver which lacks microbubble contrast correspond to area of liver laceration. The pseudoaneurysm was confirmed during conventional hepatic artery catheter angiography (**D**, arrow) and subsequently successfully embolized with vascular coils

Beside characterization of parenchymal injuries, CEUS is particularly valuable for detection of vascular abnormalities not visualized with the unenhanced B-mode US. These abnormalities include parenchymal infarcts, arterial pseudoaneurysms, and active hemorrhage [1, 56, 57] (Fig. 7). The extravasation of UCA represents foci of active hemorrhage and has been reported in splenic, liver, and renal trauma. UCA extravasation may be visualized as rounded echogenic pools or as fountain-like echogenic jets [17].

### Transplantation

US is routinely performed for monitoring of transplanted organs during the post-operative period for early detection of complications, including arterial occlusion or stenosis and venous thrombosis. The unenhanced techniques of B-mode, color Doppler, and spectral analysis are an invaluable tool for screening for these complications. However, sensitivity is limited with inconclusive results. Further imaging is often warranted either with a non-invasive type of angiography (CTA or MRA) or with interventional angiography. CEUS is well-established for the evaluation of both micro-vasculature and macro-vasculature, acting as a potential alternative to CTA or MRA.

It has been established that the administration of UCA increases the diagnostic accuracy of US for the detection of ischemic areas of both native and transplanted organs, demonstrating areas of ischemia with increased tissue contrast, depicted as non-enhancing areas within the normally perfused parenchyma. Color Doppler provides only a subjective assessment of tissue vascularity based on the color flow signals [58–61].

**Fig. 8:**
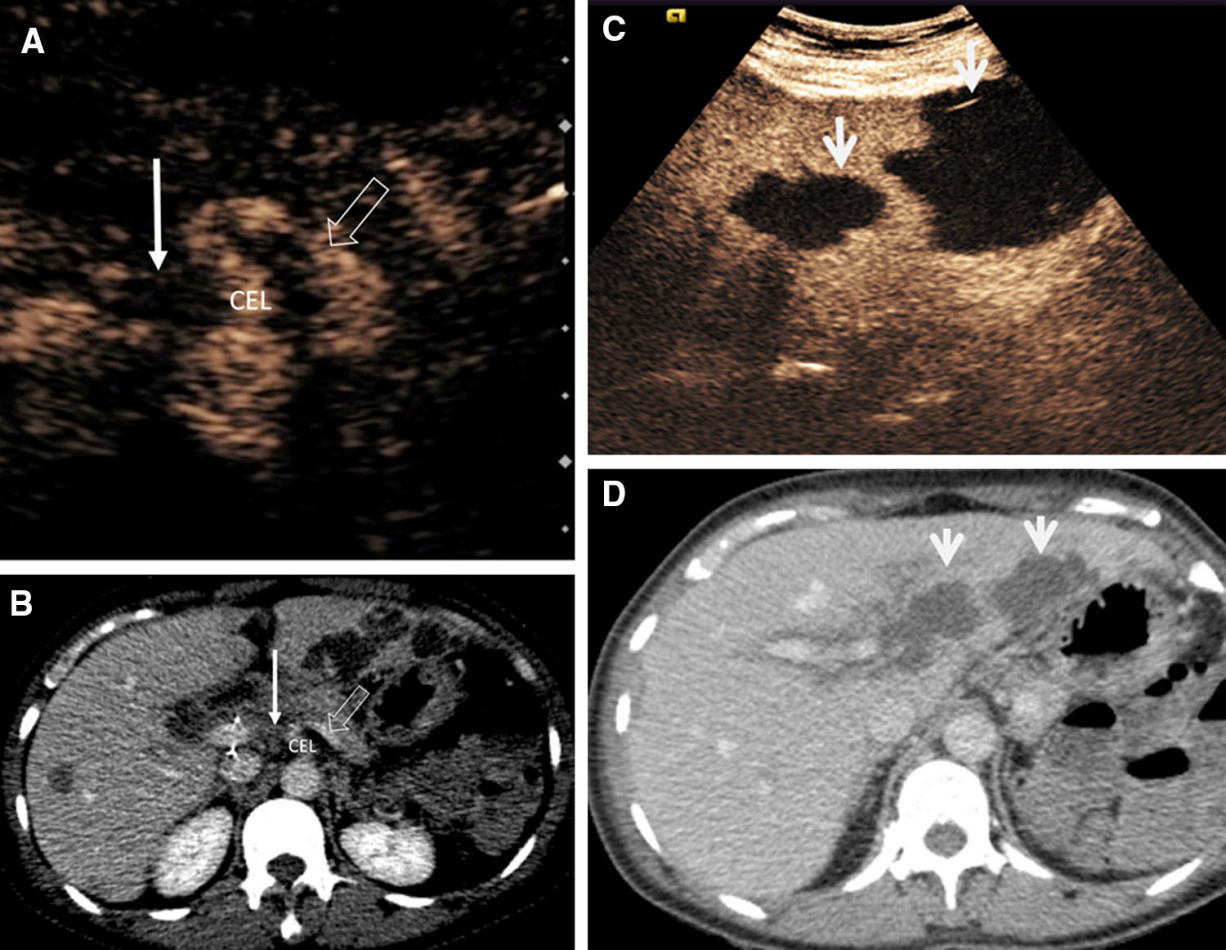
A 60-year-old female developed hepatic artery thrombosis post-liver transplantation. The CEUS (**A**) and axial CECT (**B**) comparison images demonstrate proximal hepatic artery thrombosis (solid arrow) with lack of microbubble ultrasound contrast or iodinated contrast vascular enhancement. The coeliac trunk is labeled as CEL and the splenic artery is indicated by open arrows. There is widespread resultant geographical areas of hepatic infarction present on the CECT and CEUS images (arrows, **C** and **D**)

CEUS is useful for assessment of liver transplantation complications including hepatic artery and portal vein occlusion and stenosis, active hemorrhage, pseudoaneurysm formation, and parenchyma infarction. The intravenous administration of UCA provides real-time evaluation of tissue perfusion and detailed vascular opacification, offering greater confidence for the performing physician and facilitating the visualization of patent, occluded, or stenotic hepatic arteries (Fig. 8). CEUS may obviate the need for interventional angiography in > 60% of patients [62–66] and is 92.3% sensitive and 87.5% specific for the diagnosis of hepatic artery stenosis, correcting false-positive results found on color Doppler. CEUS may be introduced in a diagnostic algorithm following color Doppler US for evaluation of liver transplant vasculature, with positive findings on CEUS warranting investigation with angiography [67].

CEUS following renal transplantation can be used to identify acute cortical necrosis, demonstrating the peripheral rim sign, as seen on CT and MR imaging [68]. Functional information related to the transplanted kidney can be obtained with quantification of parenchymal signal intensity on CEUS. This technique produces time–intensity curves and dynamic variables like time-to-peak and peak intensity. Good inter-observer agreement was demonstrated with this type of analysis, while the quantitative variables obtained have been correlated with glomerular filtration rate 3 months after transplantation [69].

### Other abdominal vessels

**Fig. 9:**
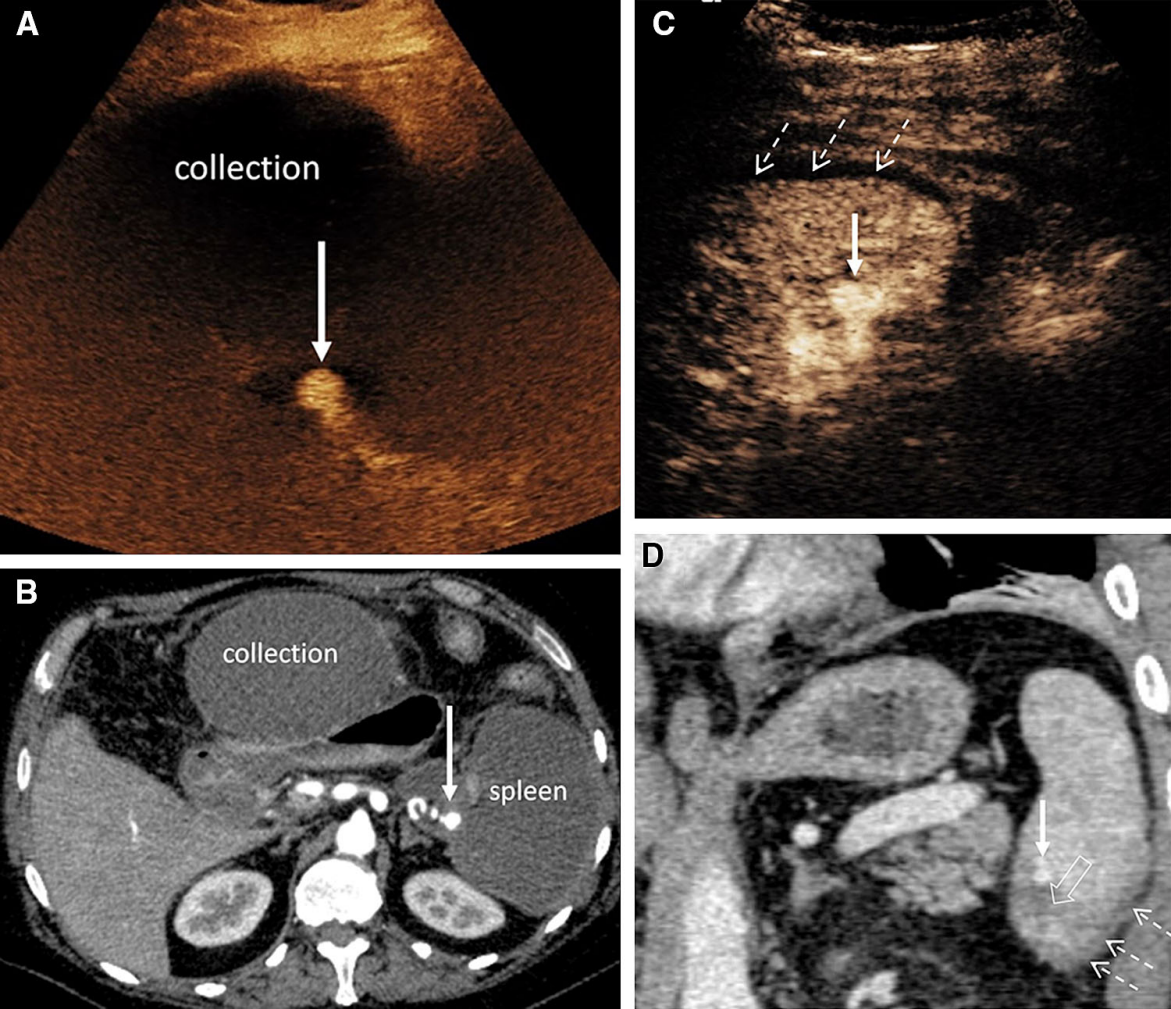
CEUS and CECT images from two cases of splenic artery pseudoaneurysm formation (arrows). **A** (CEUS) and **B** (CECT, axial) from a 47-year-old male who developed splenic artery pseudoaneurysm encased by necrotising pancreatitis with large peripancreatic collection and completely infarcted non-enhancing spleen. **C** (CEUS) and **D** (CECT, coronal) from a 43-year-old male who suffered blunt abdominal trauma demonstrates a small peri-splenic hematoma (broken arrows) and a pseudoaneurysm (arrow) adjacent to the laceration (open arrow)

**Fig. 10:**
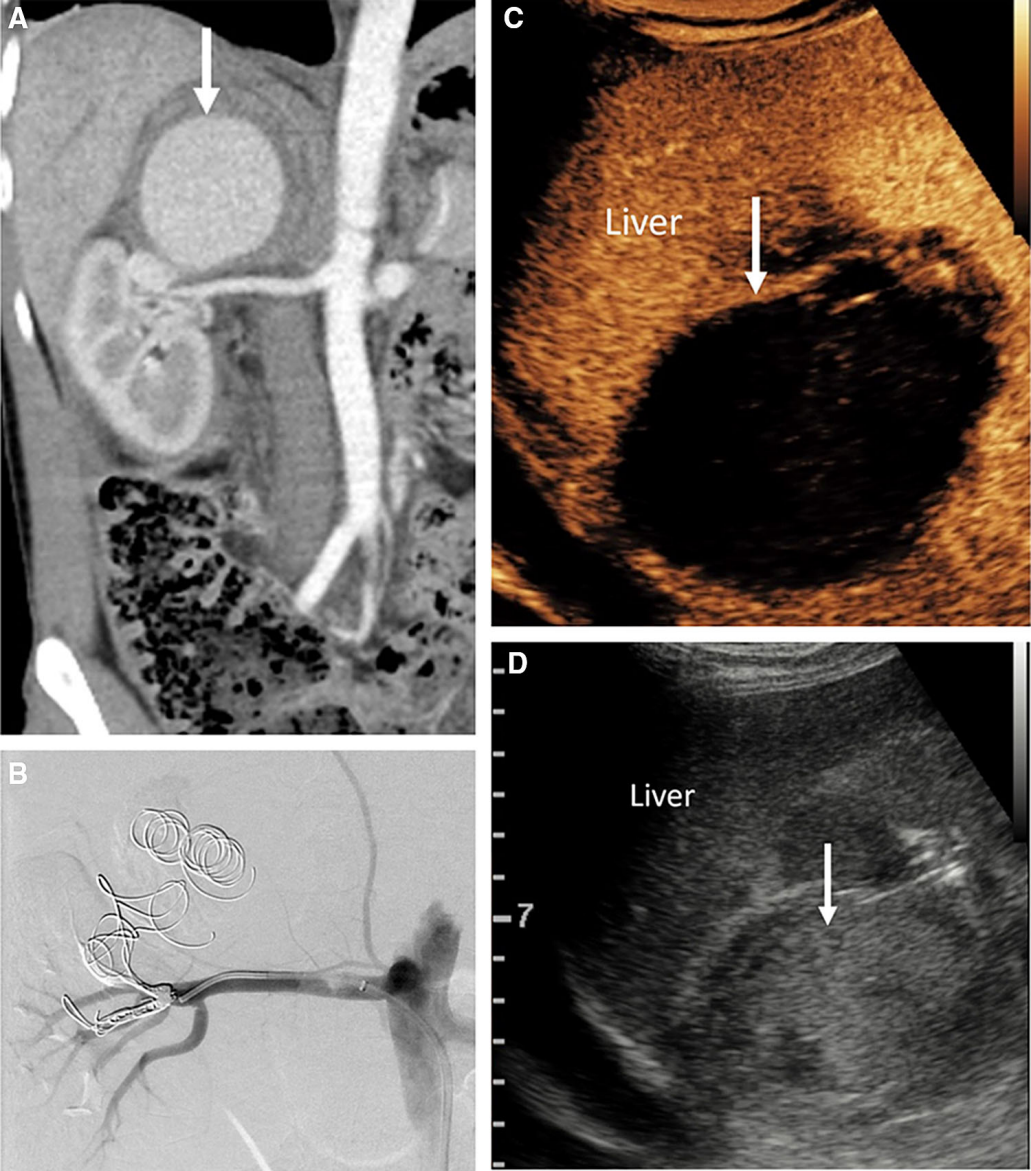
Images from a 40-year-old male with suspected renal colic. Coronal CECT (**A**) shows a giant branch renal artery pseudoaneurysm (arrow) which was coil embolized (**B**). Post-procedural follow-up CEUS image (**C**) demonstrates the absence of microbubble ultrasound contrast within the aneurysm sac (arrow) in keeping with complete occlusion of the pseudoaneurysm. Grey-scale image shows echogenic thrombus within the pseudoaneurysm (**D**, arrow)

Renal and mesenteric arteries represent a challenging arterial system for US evaluation due to their deep location, tortuous course, and overlying bowel gas. Recent technological advances and widespread availability of CTA or MRA have replaced interventional angiography for evaluation of renal and mesenteric artery stenosis. CEUS is able to accurately detect and delineate aneurysms affecting virtually any blood vessels visualized with US (Fig. 9). Moreover, CEUS may play an important role in the post-interventional management of such patients; identifying residual flow within aneurysms or establishing their successful embolization (Fig. 10). The use of UCA may be expected to increase accuracy for evaluation, but there is limited literature available [1].

**Fig. 11:**
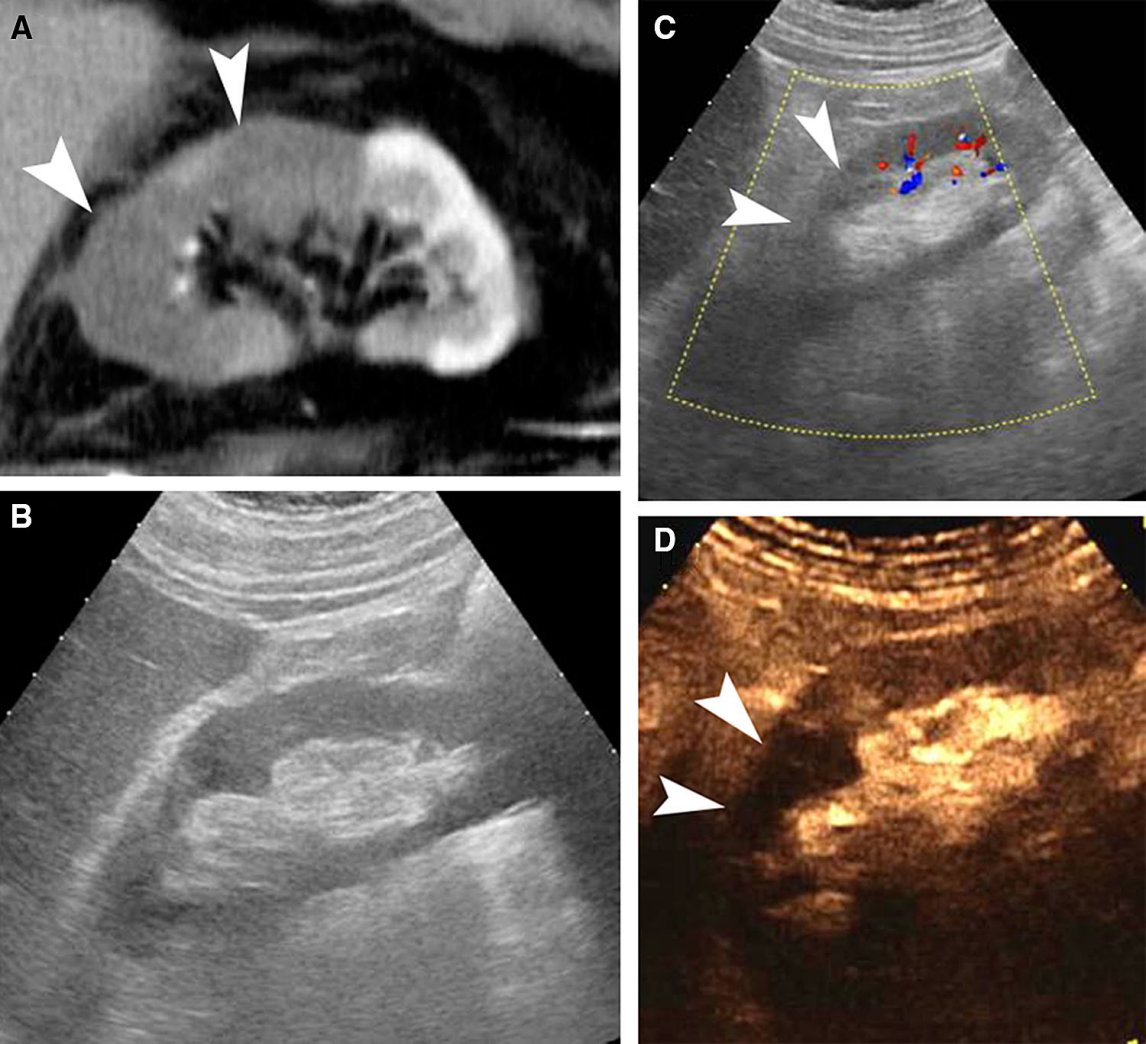
Imaging findings in a patient with renal infarction. Oblique sagittal MDCTA image (**A**) showing the kidney in long-axis, revealed the presence of an upper pole renal infarct (arrowheads). On follow-up US, long-axis B-mode technique (**B**) did not visualize any significant alterations in parenchymal echogenicity. Respective color Doppler technique image (**C**) demonstrated less blood flow signals on the upper half of the kidney (arrowheads), in keeping with the infarct. Respective CEUS image (**D**) readily confirmed the diagnosis of renal infarction (arrowheads)

The administration of Levovist™ (Schering, AG, Berlin), a first-generation UCA, allowed for quicker evaluation of intrarenal arteries Doppler spectrum with superior sensitivity and specificity compared to the unenhanced technique [70]. The administration of UCA can be used to aid correct placement of the sample volume during pulsed-wave Doppler interrogation of the renal arteries, increasing the technique’s sensitivity [71]. Similarly, CEUS with Levovist™ was also found superior to the unenhanced color Doppler technique, showing excellent agreement with interventional angiography for grading renal artery stenosis [72]. Evaluation of renal parenchyma perfusion with CEUS is superior to the unenhanced color Doppler technique and almost equivalent to contrast-enhanced CT, based on the increased tissue contrast achieved between viable and ischemic tissue, primarily a consequence of the truly intravascular nature of the UCA [1, 58]. Renal parenchymal infarction appears on CEUS as hypoechoic non-enhancing wedge-shaped areas, readily differentiated from cortical necrosis [1, 59] (Fig. 11).

The use of UCA for the evaluation of other aortic branches is limited to the superior and inferior mesenteric artery. Definity™ (Lantheus Medical Imaging, Billerica, Massachusetts) offers increased sensitivity for the identification of celiac and mesenteric artery stenosis and occlusion [73]. Others have investigated the use of UCA for evaluation of mesenteric transit time in Crohn’s disease, and even though visual and software-based assessment of the time of maximum UCA enhancement in the superior mesenteric artery and vein correlated well, there was no significant correlation with disease activity [74].

CEUS has also been used to detect liver metastasis by evaluating hepatic artery and vein enhancement based on the arrival times to hepatic artery and vein and transit time between artery and vein; shorter with an increased level of enhancement in both vessels in patients with liver metastases. Based on these results, a functional ultrasonographic technique performed with only 0.6 mL of SonoVue™ can be used to detect micrometastases in the liver [75].

## Conclusion

The introduction of UCA has significantly expanded the role of US in the investigation of abdominal vascular diseases. CEUS is superior to conventional US techniques in term of tissue contrast, spatial, and temporal resolution and its dynamic and real-time nature in assessment of tissue perfusion and vascular lumen opacification. Experience has shown that CEUS plays a key role in certain clinical scenarios such as evaluation of abdominal trauma, diagnosis of organ ischemia, imaging surveillance of post-EVAR aorta or the differential diagnosis of malignant vs. benign portal vein thrombosis in patients with liver cirrhosis and hepatocellular carcinoma. CEUS is also useful in assisting ultrasonographic evaluation of other blood vessels, although the widespread availability of CTA and MRA has limited its role in the renal arteries and mesenteric arteries.

## Electronic supplementary material

Below is the link to the electronic supplementary material.

**Online Resource 1** Video clip of CEUS examination showing an endoleak type 1. Supplementary material 1 (AVI 446553 kb)

**Online Resource 2** Video clip of CEUS examination showing an endoleak type 2. Supplementary material 2 (AVI 66479 kb)

**Online Resource 3** Video clip of CEUS examination showing an endoleak type 3. Supplementary material 3 (RAR 314797 kb)
